# Living cumulative network meta-analysis to reduce waste in research: A paradigmatic shift for systematic reviews?

**DOI:** 10.1186/s12916-016-0596-4

**Published:** 2016-03-29

**Authors:** Per Olav Vandvik, Romina Brignardello-Petersen, Gordon H. Guyatt

**Affiliations:** Department of Medicine, Innlandet Hospital Trust, Gjøvik, Norway; Norwegian Knowledge Centre for the Health Services, University of Oslo, PB 7004 St.Olavsplass, 0130 Oslo, Norway; Department of Clinical Epidemiology & Biostatistics, McMaster University, Hamilton, Ontario Canada; Evidence-Based Dentistry Unit, Faculty of Dentistry, Universidad de Chile, Santiago, Chile

**Keywords:** Systematic reviews, Network meta-analysis, Evidence-based medicine, Knowledge translation, Clinical practice guidelines, GRADE

## Abstract

In a recent research article in *BMC Medicine*, Créquit and colleagues demonstrate how published systematic reviews in lung cancer provide a fragmented, out-of-date picture of the evidence for all treatments. The results and conclusions drawn from this study, based on cumulative network meta-analyses (NMA) of evidence from randomized clinical trials over time, are quite compelling. The inherent waste of research resulting from incomplete evidence synthesis has wide-reaching implications for a range of target groups including developers of systematic reviews and guidelines and their end-users, health care professionals and patients at the point of care. Building on emerging concepts for living systematic reviews and NMA, the authors propose "living cumulative NMA" as a potential solution and paradigmatic shift. Here we describe how recent innovations within authoring, dissemination, and updating of systematic reviews and trustworthy guidelines may greatly facilitate the production of living NMA. Some additional challenges need to be solved for NMA in general, and for living cumulative NMA in particular, before a paradigmatic shift for systematic reviews can become reality.

Please see related research article: https://bmcmedicine.biomedcentral.com/articles/10.1186/s12916-016-0555-0

## Background

Health care professionals and other decision makers responsible for providing patients with safe and high quality care need access to the best current research evidence about diagnosis, treatment and prognosis [1]. High quality systematic reviews synthesize the best available evidence regarding an intervention’s benefits and harms to answer a clinical question. Clinical practice guidelines combine this evidence with other relevant considerations such as patients’ values and preferences [2]. Together, reviews and guidelines constitute key tools to provide the best current evidence and optimal recommendations for choosing among alternative diagnostic and treatment strategies [3].

Over the past decades the world has seen large advances in standards, methods, and systems for conducting and reporting systematic reviews and trustworthy guidelines [4–7]. These advances include systematic reviews that use network meta-analyses (NMA), the statistical technique that allows comparing multiple treatments at a time [8, 9], the methods of which are rapidly evolving [10].

In a study recently published in *BMC Medicine*, Créquit and colleagues illustrate how published systematic reviews in lung cancer provide a fragmented, out-of-date picture of the evidence for all treatments [11]. The evidence covered by existing systematic reviews for second-line treatments for advanced non-small cell lung cancer was incomplete, with 40 % or more of treatments, treatment comparisons, and trials missing. The incomplete evidence was detected through the construction of cumulative networks of evidence from randomized clinical trials over time and an evaluation of the proportion of trials, patients, treatments, and treatment comparisons not covered by systematic reviews on a yearly basis from 2009 onwards.

The study has limitations, including lack of consideration of the scope of the systematic reviews (limited scope may have been a legitimate reason to not include all available trials), and failure to address the possibility that it would have been inappropriate to pool together the results of all the randomized trials detected. Although these limitations may have resulted in an exaggeration of the magnitude of the problem, the results and conclusions are nevertheless compelling. As stated by the authors "available pairwise treatment comparisons do not allow for meeting clinicians’ and patients’ needs in decision making. Nor do they meet the needs of other target audiences such as developers of clinical practice guidelines, decision aids, funders and decision-makers in health care systems" [11].

### Living cumulative NMA

Building on emerging concepts for living systematic reviews and network meta-analysis, the authors conclude that this waste of research might be reduced by "living cumulative NMA" (living NMA hereafter). With this approach, the focus would be on developing NMAs that include all available treatments (as opposed to a series of head-to-head comparisons), which are updated as soon as new trials become available. The authors propose methodological steps for living NMA and present a series of challenges and potential solutions for each step from creation to dissemination and updating. Their proposed solution represents an innovative input to existing challenges for systematic reviews and also relates to international calls for increased value and reduced waste in research, here within the context of evidence synthesis of available trials [12].

Although producing living NMAs is challenging, recent developments could greatly facilitate their production. Improved methods, strategies, and tools for more efficient authoring, dissemination, and updating of systematic reviews and trustworthy guidelines are emerging [13–15]. Tools, such as the Epistemonikos database, allow efficient and intuitive searching for systematic reviews and individual studies [16]. The use of GRADE combined with innovative health information technology (e.g., MAGICapp) facilitates dynamically updated digitally structured evidence summaries, guidelines, and decision aids that clinicians and patients can access on all devices in user-friendly formats [3, 14, 15, 17, 18]. Presentations on these devices include desirable and undesirable consequences of treatment alternatives customized for both shared decision-making and integration in electronic medical records linked to patient specific data [14, 19].

These innovations, however, have not fully addressed all the challenges related to NMA in general, and to living cumulative NMA in particular. We will now comment on some additional challenges and suggest potential solutions from the perspective of developers of systematic reviews and guidelines and their end-users, health care professionals and patients at the point of care.

### Rating quality of evidence in NMA

Systematic reviews need to perform an appropriate assessment of the quality of the body of evidence (synonyms: certainty or confidence in the evidence) for patient-important outcomes [5, 7]. State of the art reviews now provide evidence summaries using GRADE Summary of Findings (SoF) tables that provide quality of evidence ratings to inform clinicians, patients, guideline developers, and policy decision-makers [20].

Créquit and co-authors point out that guideline developers and other decision-makers may further benefit from NMAs if they rate the quality of the body of evidence supporting treatment effect estimates for all patient-important outcomes. Rating quality of evidence in NMA presents additional challenges and represents a methodological frontier. Authors have already presented two systems based on GRADE principles for making quality of evidence ratings in NMAs [21, 22]. Much work remains in the testing, application, and refinement of these methods, currently being undertaken by (among others) the GRADE NMA project group [23].

### Patient-important outcomes in NMA

Systematic reviews and trustworthy guidelines need to provide evidence for all outcomes to allow an assessment of the balance between the desirable and undesirable consequences of a course of action [4, 24]. The Créquit study focused on the availability of treatment comparisons for lung cancer rather than on the extent to which authors synthesized evidence across all patient-important outcomes. As acknowledged by the authors, the identification and reporting of all outcomes that are important to patients for decision-making in living NMA represents a particular challenge, including differential reporting across outcomes resulting in variation in the geometry of the network of trials. Further methodological work in NMA will be required to address these issues.

### Dissemination of results from NMA to the point of care

Health care professionals and patients will, as outlined above, increasingly be able to access the best current evidence from systematic reviews and recommendations in guidelines at the point of care in new and user-friendly formats [3, 14, 19]. Interactive Summary of Findings (iSoF) tables represent one such innovation with new and improved presentation formats of evidence summaries linked to systematic reviews and guidelines. iSoF tables allow end-users to balance benefits and harms in absolute numbers, also taking into account the quality of the evidence [25].

Current SoF tables are structured only for paired comparisons, and so do not address the multiple comparisons of NMA. How to best present multiple comparisons for all patient-important outcomes to end-users remains uninvestigated. Members of the GRADE NMA project group are exploring both how to optimally present the results from NMA in SoF tables and how to translate relative estimates of effects (the output obtained from NMA) into absolute estimates of effect that facilitate the decision-making process [23].

## Conclusions

The traditional methods of conducting systematic reviews are associated with a risk of presenting an incomplete picture of the relevant evidence addressing a clinical question. Living NMA represents a promising approach to providing a broad, complete, and updated presentation of the evidence regarding all the available management options. This might well represent a paradigmatic shift for systematic reviews. However, as is the case for any new technology, living NMA will require overcoming a number of challenges. Investigators are currently addressing these challenges which suggest that living NMA as a new tool for addressing clinical questions may, in the near future, become available.
